# Novel selective, potent naphthyl TRPM8 antagonists identified through a combined ligand- and structure-based virtual screening approach

**DOI:** 10.1038/s41598-017-11194-0

**Published:** 2017-09-08

**Authors:** Andrea R. Beccari, Marica Gemei, Matteo Lo Monte, Nazareno Menegatti, Marco Fanton, Alessandro Pedretti, Silvia Bovolenta, Cinzia Nucci, Angela Molteni, Andrea Rossignoli, Laura Brandolini, Alessandro Taddei, Lorena Za, Chiara Liberati, Giulio Vistoli

**Affiliations:** 1 Dompé Farmaceutici SpA, Via Campo di Pile, L’Aquila, L’Aquila, Italy; 2 Axxam S.p.A, Via Meucci, 3, I-20091 Bresso, Italy; 3 Dipartimento di Scienze Farmaceutiche, Università degli Studi di Milano, Via Mangiagalli, 25, I-20133 Milano, Italy; 4 Joint Bioinformatics Group, Institute of Protein Biochemistry; National Research Council, Via P. Castellino 111, Napoli, Italy

## Abstract

Transient receptor potential melastatin 8 (TRPM8), a nonselective cation channel, is the predominant mammalian cold temperature thermosensor and it is activated by cold temperatures and cooling compounds, such as menthol and icilin. Because of its role in cold allodynia, cold hyperalgesia and painful syndromes TRPM8 antagonists are currently being pursued as potential therapeutic agents for the treatment of pain hypersensitivity. Recently TRPM8 has been found in subsets of bladder sensory nerve fibres, providing an opportunity to understand and treat chronic hypersensitivity. However, most of the known TRPM8 inhibitors lack selectivity, and only three selective compounds have reached clinical trials to date. Here, we applied two virtual screening strategies to find new, clinics suitable, TRPM8 inhibitors. This strategy enabled us to identify naphthyl derivatives as a novel class of potent and selective TRPM8 inhibitors. Further characterization of the pharmacologic properties of the most potent compound identified, compound 1, confirmed that it is a selective, competitive antagonist inhibitor of TRPM8. Compound 1 also proved itself active in a overreactive bladder model *in vivo*. Thus, the novel naphthyl derivative compound identified here could be optimized for clinical treatment of pain hypersensitivity in bladder disorders but also in different other pathologies.

## Introduction

Transient receptor potential (TRP) channels are a group of ion channels located primarily on the plasma membrane of numerous animal cell types^1,2^. These channels are characterized by six transmembrane subdomains flanked by intracellular C- and N-terminal regions, and they are capable of homo- or hetero-tetramerization to form cation-permeable pores^3^. These channels mediate responses to a wide range of stimuli, such as pain, heat, warmth or coldness, as well as taste, pressure, and vision. Members of the mammalian TRP channel family can be subdivided into seven classes according to their sequence homology: TRP ankyrin (TRPA), TRP canonical (TRPC), TRP melastatin (TRPM), TRP melastatin-like (TRPML), TRP NOMPC (TRPN), TRP polycystic (TRPP) and TRP vanilloid (TRPV). Eight of the family members are recognized as thermo-TRPs in that they are expressed in primary somatosensory neurons and are activated at specific temperatures ranging from noxious heat to painful cold. TRPV1–4 transduce elevated temperatures ranging from warm (TRPV4 and TRPV3) to noxious heat (TRPV1 and TRPV2). By contrast, TRPM8 and TRPA1 are activated by moderate and more extreme cooling, respectively. All thermo-TRPs are also activated by a wide range of natural compounds. TRPM2, TRPM4 and TRPM5 also show temperature sensitivity but are not usually included in the thermo-TRP family because they are not expressed in primary somatosensory neurons^4^. TRP channels are not particularly closely related by either structure or function. In fact, the most similar TRPM channel to TRPM8 is TRPM2, but this channel is involved in oxidative stress sensing and is expressed in dopaminergic neurons and other tissues in which TRPM8 is not present^5,6^.

Transient receptor potential melastatin type 8 (TRPM8) is a member of the transient receptor potential (TRP) superfamily^2^. More specifically, TRPM8 is a ligand-gated, nonspecific cation channel that is activated by cold temperatures, by natural cooling compounds, such as menthol and eucalyptol, and by synthetic agents, such as icilin^7^.

TRPM8 expression defines a population of sensory afferents that innervate tissues known to be highly sensitive to cold stimuli, as well as nociceptive stimuli^8^. In addition, the localization of TRPM8 on nociceptive A-delta and C-fiber neurons and its modulation by inflammation-mediated second messenger signals, may account for abnormally cold sensitivity in some pathologic states, providing a good molecular basis and a rationale for the design of TRPM8 antagonists^9^. Indeed, characterization of TRPM8 has revealed its role in pain hypersensitivity^10,11^ that is manifested in two forms: cold allodynia and cold hyperalgesia^12^. Cold allodynia refers to pain induced by a normally innocuous cold temperature, and is one of the main clinical symptoms experienced by patients undergoing chemotherapy. It can also occur in patients with other conditions such as diabetic neuropathy^13^, fibromyalgia, and traumatic neuropathy^11^. Cold hyperalgesia, in which a noxious stimulus results in a prolonged or intense pain response, commonly occurs with chronic inflammation^14^, such as in rheumatoid arthritis^15^. During the time, many other painful conditions have been related to TRPM8 activity. The recent discovery of different receptors in the bladder, in subsets of sensory nerve fibres, responsive to temperature or molecules as capsaicin and menthol provides an opportunity to understand and treat TRPM8-related bladder painful disorders. For example, TRPM8 has been related to the painful or overactive bladder syndromes (OBS) ad painful bladder (PBS) or overreactive bladder syndromes^16^. PBS is a chronic bladder hypersensitivity disorder which causes suprapubic pain along with an increased micturition frequency and nocturia, while OBS is characterized by urinary urgency with or without urge incontinence and increased frequency^17^. Additionally, an immunohistochemical study has shown that the density of the TRPM8 channel protein is elevated in bladder afferent nerves in overactive bladder patients compared with normal subjects^18^. Moreover, there is a positive correlation between TRPM8 density and voiding frequency^18^. Altogether these findings suggested that TRPM8 could be a promising target to develop drugs to treat different painful disorders^19,20^. Known TRPM8 antagonists primarily belong to the chemical classes of para-menthane-based or non-para-menthane-based ligands^21–23^, depending on the presence of the menthol scaffold. Many of these TRPM8 antagonists, such as BCTC^24^, CTPC^25^, and capsazepine^26^ are also antagonists of other TRP channels, particularly TRPV1 and TRPA1, thus suggesting a conserved mechanism for the ligand activation of these thermosensitive TRP channels^27–30^. This overlapping mechanism of activation makes the identification of selective agents for these ion channels difficult. To date, only three TRPM8 antagonists have reached clinical evaluation: the quinolone- and pyridine-carboxamide derivatives PF-05105679 (Pfizer; ClinicalTrials.gov Identifier: NCT01393652) and AMG-333 (AMGEN; ClinicalTrials.gov Identifier: NCT01953341), which entered but did not pass phase I studies^30,31^, and the cannabinoid Cannabidivarin (GWP-42006) (GW Pharmaceuticals; ClinicalTrials.gov Identifier: NCT02365610), which is currently in phase II clinical study^31,32^. Thus, the need for novel TRPM8 inhibitors is urgent and many companies and researchers are working on this target.

Currently, virtual screening plays a key role in the discovery of novel drugs, representing a cost-effective and efficient method to increase hit rates compared to wet-lab high-throughput screening (HTS)^33–36^. Virtual screening techniques can be subdivided into ligand-^37,38^ and structure-based^39,40^ approaches; the former is mainly based on similarity analyses and the latter mostly involves docking simulations. Recent studies have indicated the possible fruitful combination of both methods^41^.

Here, with the aim to discover novel TRPM8 inhibitors, we used a combination of ligand- and structure-based approaches to screen a proprietary library of 124,107 highly diverse compounds. Promising active molecules (a total of 11,725) were then subjected to conventional wet-lab screening using the calcium-sensitive fluorescent dye approach^42,43^. This approach enabled the identification of a set of naphthyl derivatives as new potent and selective TRPM8 inhibitors (US8906946, Dompe’ SpA 2014). Among this set, compound 1 was identified as a selective TRPM8 inhibitor, with potency in the nanomolar range and a competitive mode of action. We tested compound 1 in preliminary *in vivo* tests using an isovolumetric bladder rat model to assess its possible usefulness in painful and overreactive bladder syndromes^44^. We observed that compound 1 had a positive effect on inhibition time, threshold bladder volume-inducing rhythmic bladder contraction (RBC) and micturition frequency (MF). Taken together, our results suggest that compound 1 could be a good candidate for the development of clinical suitable drugs for the treatment of urologic disorders as PBS or OBS.

## Methods

### Pharmacophore mapping

**Figure 1:**
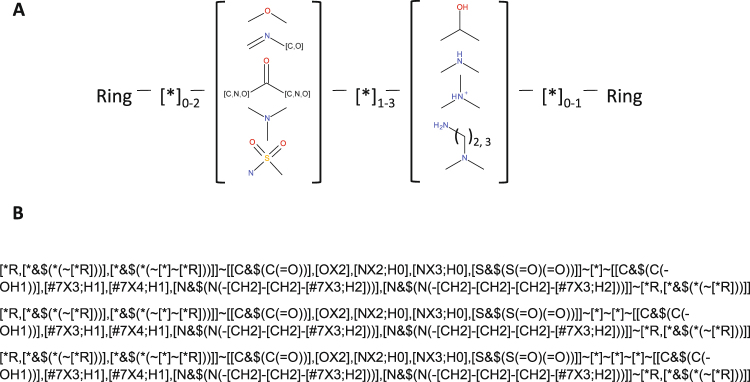
Derived pharmacophore through SMARTS strings. (**A**) Schematization of the derived pharmacophore model. (**B**) SMARTS strings encoding for the pharmacophore.

Pharmacophore feature extraction was performed using the Smiles Arbitrary Target Specification (SMARTS) language^45^ to encode the structure-activity relationship of a selection of structurally unrelated TRPM8 antagonists, as schematized in Fig. 1. SMARTS queries are commonly used in the definition of molecular motifs and have a wide application in sub-structures filtering, such as flagging toxicological structures in library screening^46^. First, SMARTS language was used to encode the structure-activity relationship on a selection of structurally unrelated TRPM8 antagonists, as schematized in Fig. 1. Subsequently, SMARTS queries were extended to include additional chemical motifs not represented in the reference set and to exclude undesired chemical moieties. Together, these derived SMARTS queries were used to virtually screen a proprietary database and to generate a ligand-based targeted library of putative TRPM8 antagonists.

To date, approximately 120 different chemotypes can be enumerated among all known TRPM8 antagonists (as defined by Bemis *et al*.^47^). To represent this wide chemical spectrum, 12 structurally diverse TRPM8 antagonists were selected for pharmacophore development, each reporting on a characteristic structural moiety and spacing in molecular flexibility and ring heterogeneity (size and complexity), as well as accounting for different polar groups (Figure S1). As shown schematically in Fig. 1A the resulting pharmacophore consisted of a hydrogen bond acceptor system (HBA) and a hydrogen bond donor system (HBD) between two terminal rings with variable length spacers separating each functional group (Fig. 1B). This general motif, in addition to covering the 12 selected antagonists, included approximately 50 of the abovementioned TRPM8 antagonist chemotypes.

**Figure 2:**
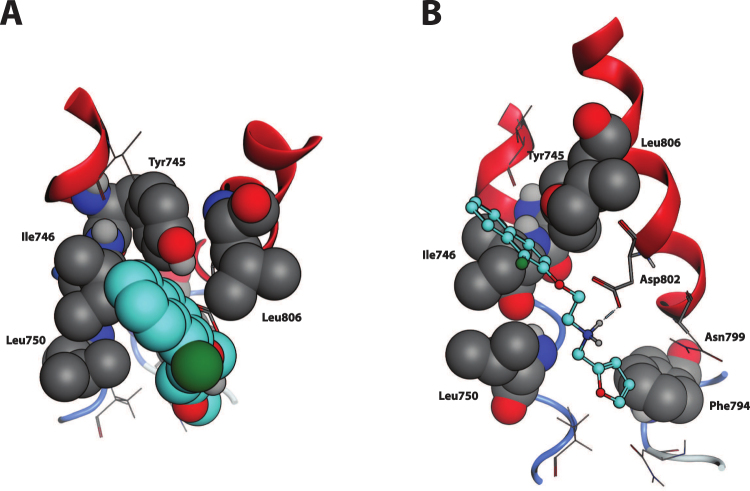
TRPM8-Compound 1 complex, detail of the binding site. (**A**) Top view, perpendicular to the membrane plane, extra- to intracellular perspective. The naphthyl moiety of the ligand, here highlighted in cyan and represented in CPK, well fits into the sub-pocket framed by Ile746, Tyr745, Leu750 and Leu806 (in grey and CPK). (**B**) Side view, perpendicular to the transmembrane helices. Compound 1, represented in cyan and ball-end-stick, lands parallel to TM helices 2 and 3 surface, here in red cartoon. Dotted blue line represents the Hydrogen bond between the amine group of Compound 1 and the carboxyl function of Asp802, interaction further strengthened by opposite charge attraction. The furane ring locates between Leu750 and Phe794 (in grey and CPK).

In more details, the HBA group is represented by direct and reverse sulfonamide, ether, ester, ketone and imine groups, whereas the HBD group is represented by amido, amino and hydroxyl groups. In the twelve selected TRPM8 antagonists (Figure S1), the two H-bonding groups can be spaced by the following number of atoms: (*i*) one atom, as in the case of compounds 1, 6, 8, 10 and 11; (*ii*) two atoms, as for compounds 3, 5, 9 and 12; or (*iii*) three atoms, as in compounds 2 and 7. This pattern, which condenses the structure-activity relationships among the active hits, can be encoded by three SMARTS strings (Fig. 1B). Considering the constrained systems it has been proposed that the *cis* orientation is preferred, which is consistent with the proposed binding mode for TRPM8 inhibitors on the basis of the derived 3D homology models of the ion channel (Fig. 2). One of the 12 antagonists selected and presented in Figure S1, the fourth, by Bayer, despite having five atoms separating the two H-bonding functions, was included in the set because the high flexibility of the ethylamine chain (HBD) can approach the *para*-ether group (HBA), thus “spatially” matching the pharmacophore. Up to two atoms are allowed between the terminal ring and the HBA group, whereas a maximum of one atom is permitted between HBD and the other ring. Either aromatic or aliphatic rings are allowed and can include the H-bonding function.

To further enlarge the selection to encompass different scaffolds, the pharmacophore was extended to include other HBA or HBD groups. Additionally, a collection of more than 200,000 biologically relevant compounds from the MDDR Database^48^ which are already annotated for their chemical structure, physicochemical properties and biological activity. This collection was then analyzed to identify chemical moieties sharing the same pharmacophore, although with different biological activities and different chemotypes. Chemical moieties with undesired mechanisms of action (or specific targets) were excluded. Using the same rationale used to define the pharmacophore model, a total of 37 SMARTS strings related to the undesired moieties were defined (seven representative SMARTS are presented in Table S2). After the optimal set of SMARTS strings was defined, they were used to screen the corporate library to identify TRMP8 blockers while discarding nonselective or undesired scaffolds and maximizing chemical diversity.

### Structure-based virtual screening strategy optimization

We used already known TRPM8 inhibitors to develop a docking-based screening strategy on the TRPM8 receptor structure. For this purpose, we collected a benchmarking set comprising 50 published TRPM8 antagonists^22^ and 4,950 decoy compounds taken from known inactive compounds, as identified in previous HTS campaigns (data not shown). To avoid bias in the results, inactive compounds were selected only if they fulfilled several physicochemical property ranges, as determined by analyzing the active compounds.

The obtained compound set was used in preliminary docking simulations on a previously published human TRPM8 homology model^49^. We used a homology model generated by fragments of the resolved structure of the Kv1.2 Shaker channel as the overall template^50^. To date, the only additional structure available is the recently resolved TRPV1 structure^51^; however, TRPV1 does not have a significantly high degree of similarity with TRPM8 with respect to our model (pairwise alignment BLOSUM62 score 0.39 vs 0.35)^52^. Moreover, TRPV1 structure allows for reliable generation of only the transmembrane bundle. The Kv1.2 Shaker channel remains reliable for modeling the binding site within the transmembrane bundle in an unbound conformation, as supported by the positive results obtained in previous publications^45,49,52–54^ as well as by the satisfactory docking results reported here. Both the benchmarking set and the virtually screened library were prepared using an automatic script in the VEGAZZ suite of programs^55^, which performs the following tasks for each compound: (*i*) generating the 3D structure; (*ii*) adding the hydrogen atoms; (*iii*) assigning the atom types and the Gasteiger’s atomic charges; (*iv*) selecting the predominant form of ionizable molecules at physiological pH; (*v*) generating all possible stereoisomers for chiral molecules; and (*vi*) minimizing the obtained molecules by combining steepest descent and conjugate gradient algorithms.

Docking simulations were performed using the LiGen^50,56^ program and were optimized by tuning the arrangement and the extension of the box where the docking search was performed. Note that the docking parameters of the program were not adjusted because they had already been optimized by experimental design in a previous study, thus maximizing virtual screening performance^50^. Before performing docking simulations, LiGen requires the development of a suitable pharmacophore model by using LiGenPocket. To this end, a relatively large and highly potent TRPM8 antagonist (Cpd 13 WO2007017094, belonging to the class of benzyloxy-phenylmethylcarbamates with an IC_50_ = 0.45 nM) not included in the benchmarking set was utilized to avoid biasing results and to develop the pharmacophore as exhaustively as possible.

**Figure 3:**
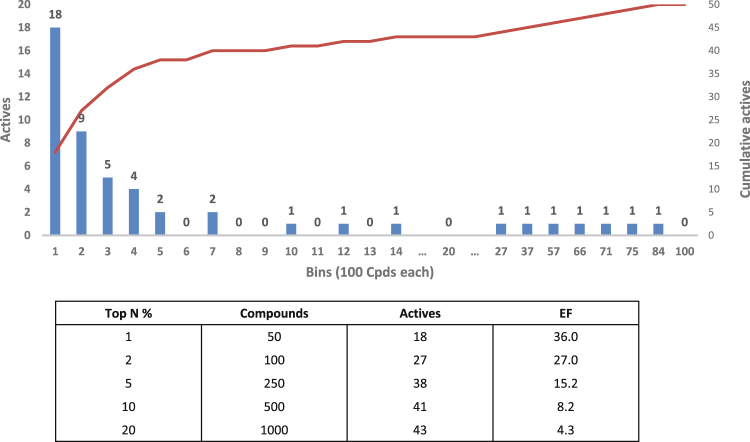
Docking results obtained by LiGen on the benchmarking dataset. Distribution of the active compounds within the entire ranking as subdivided into 100 bins, resulting from the Docking campaign targeted in an 8 Å radius sphere around Asp802. Active compounds and enrichment factor (EF) are reported for each top N %.

To the best of our knowledge, experimental evidence indicates that TRPM8 antagonists bind the menthol-binding site of TRPM8 to exert their action. Hence, the LiGen simulations were focused on the menthol-binding site and were tuned by varying the radius of the considered sphere (i.e., equal to 12, 10, 8, and 6.5 Å) and its center (around Asp802 or Tyr745) to also evaluate possible adjacent sub-pockets. The ligand was flexible, and the results were ranked on the basis of the LigenScore function. Figure 3 shows that the active compounds are satisfactorily distributed across the entire rank (subdivided into 100 bins) and that the enrichment factors obtained by the best-performing LiGen simulation involved an 8 Å radius sphere around Asp802.

### *In vitro* TRPM8 assays and high-throughput screening campaigns through analysis of intracellular calcium mobilization

All experiments were performed on HEK-293/hTRPM8 cells stably transfected with the human TRPM8 gene, except for site-directed mutagenesis analysis, which was performed on transient transfectants^57^. In details, HEK-293 cells (ATCC, Manassas, Virginia) were transfected with the pcDNA3/hTRPM8 vector by electroporation (300 Volts, 950 µF) then selected with medium containing 0,8 mg/ml G418 for 10–15 days. Then the stable transfected pool underwent two rounds of limiting dilutions, in eight 96 well plates at a cell density of 1 cell/well, in order to obtain pure clones. The best responder clone was selected based on its response to 1 mM menthol in calcium mobilization experiments. HEK-293/hTRPM8 cells were maintained in EMEM (Minimum Essential Medium Eagle with Earl’s salts Balanced Salt Solution, LONZA) supplemented with 2 mM Ultraglutamine1 (Lonza), 1% Penicillin/Streptomycin (LONZA), 10% of Fetal Bovine Serum (Euroclone) and 0,4 mg/mL G418 (InvivoGen).

For calcium mobilization assays, cells were seeded at 10,000 cells/well in 384-well plates coated with poly-D-lysine (MATRIX black/clear bottom #4332-CPL, Thermo Scientific, Waltham, MA, USA) in complete medium.Twenty-four hours after seeding, the plates were washed with Tyrode’s buffer with a Bio-Tek-ELx405 Microplate Washer (Merck Millipore; Billerica, MA, USA), and then 10 µl/well of a solution containing the fluorescent Ca^2+^ indicator Fluo-4 NW dye was added. Cells were incubated for 1 h at room temperature before test compounds were added at 3X the concentration with respect to the final wanted concentration to be tested with a FLIPR TETRA system (Molecular Devices, Sunnyvale, CA, USA). Calcium mobilization was monitored over a period of 5 min. A second injection of 10 µl/well of a reference agonist (icilin or Cooling agent 10) at 4X concentration in assay buffer was performed using the FLIPR TETRA system. Of note, for selectivity assays, the following agonists were used: 10 μM AITC (allyl isothiocyanate) for TRPA1; 100 nM Capsaicin for TRPV1; and 20 nM GSK1016790A for TRPV4.

After addition of the agonist, the fluorescence signal was recorded over the next 3 min. The bioactivity exerted by the compounds was expressed as a percentage of inhibition. The percentage scale is defined as follows: 100% inhibition was considered as the relative fluorescence units (RFUs) of the value of the MIN controls in the second injection (antagonist Capsazepine at IC_100_ = 50 µM) and 0% inhibition was considered as the RFU values of the value of the MAX controls in the second injection (Cooling agent 10 at EC_80_ = 30 µM). Data from four replicates were analyzed using GraphPad PRISM® software (version 5, GraphPad Software Inc.) to calculate the mean and standard error of the mean, to create sigmoidal concentration-response curves and to calculate IC_50_ values. Agonist and antagonist concentrations for each calcium mobilization experiment are reported in Table S1.

### Generation of TRMP8 single point mutation variants

Single point mutations were introduced in the hTRPM8-coding sequence, and the mutated TRPM8 was cloned as N-terminal c-myc tagged version into the pcDNA3 expression vector. This was done in that way that the single variations could be easily swapped from the myc-tagged expression construct into the non-tagged TRPM8 wild type expression construct.

The presence of the myc tag did not alter the functionality of TRPM8 receptor (data not shown). The list of the variants is the following: variant 1, t2233g, a2234c (=Y745A); variant 2, a2236g, t2237c (=I746A); variant 3, a2395g, a2396c (=N799A) and variant 4, a2405c (D802A). The cloning project comprised three steps. First of all, a unique XhoI restriction site was inserted by site-directed mutagenesis (SDM) using DNA of the pcDNA3_TRPM8 construct as template. The 0.6 kb DNA fragment between the unique 5′ XhoI (introduced) and 3′ BamHI (already present) restriction sites comprised all positions of the four single point mutations and could be later used for their insertion as well as swapping between myc-tagged and non-tagged TRPM8 versions. In a second step, the c-myc tag sequence was introduced embedded within the TRPM8 coding sequence by PCR primers in two partially overlapping PCRs that were then joined by bridge PCR with the external 5′KpnI and internal 3′EcoRI primers and in the last step the point mutations 1 to 4 were inserted into pcDNA3_c-myc-TRPM8 by exchanging the wild type 5′XhoI-3′BamHI fragment (0.6 kb) with each of the corresponding variation fragment derived from synthetic DNA (provided by GeneArt, ThermoFisher Scientific).The myc-tagged constructs were transiently transfected in HEK-293 cells with Lipofectamine 2000 (Invitrogen) according to the manufacturer’s protocol. The pcDNA vectors containing the wt myc-tagged TRPM8 gene and the empty pcDNA3 (mock) were separately transfected into HEK-293 cells as controls. Cells were transfected and seeded on the same day at 17,500 cells/well in poly-D-lysine-coated 384-well plates (MATRIX #4332-CPL). Twenty-four hours after seeding, the activity of transfected TRPM8 receptors was tested using the calcium mobilization assay as described above. The analysis was performed on the basis of ΔF/F° (normalized MAX-MIN), where ΔF is the MAX between time points 117 and 182 minus the MIN between time points TP114 and TP116, and F° is the basal signal at time point TP0. The obtained data from at least four wells (as replicates) were analyzed with GraphPad PRISM® software (version 5, GraphPad Software Inc.) to calculate the mean and standard error of the mean^58^, to create sigmoidal concentration-response curves and to calculate IC_50_ values.

### Cold Stimulation Experiment

The cold stimulation assay was performed as previously described^59^. Cells were seeded at 1.5 × 10^6^ cells in a T75 flask in complete medium. When cells were 80% confluent, the medium was removed, and cells were loaded by 5 ml flask of a solution of Screen QuestTM Fluo-8 NW dye (ABD Bioquest, Sunnyvale, CA, USA) added to the flask in a dark environment. Dye-loaded cell flasks were incubated for 45 minutes at room temperature in the dark. The Fluo-8 NW solution was then removed, and 10 ml of Tyrode’s buffer (5 mM KCl, 130 mM NaCl, 2 mM CaCl_2_, 5 mM NaHCO_3_, 1 mM MgCl_2_, 20 mM HEPES, pH7.4) was then added to the flasks; cells were detached by pipetting, counted by a cell counter, and then centrifuged and re-suspended in Tyrode’s buffer at 5 × 10^6^ cells/ml.

Cells were then seeded in 96-well assay plates (MicroAmpTM Optical 96-Well Reaction Plates by Applied Biosystems, SG51811-5A, Part No. N801-0560) at 100,000 cells/well; test compounds at 5X concentration were manually added to the wells at a 5 µl volume, and the assay plates were incubated at room temperature for 5 minutes; then, the experiment was performed using an ABI Prism® 7900HT Sequence Detection System (Life Technologies, USA).

The assay plates were initially held at 25 °C inside the instrument for 5 minutes, and the signal was recorded for 2 minutes. The temperature was subsequently lowered to sub-physiological levels, and the signal was recorded for 3 minutes. The raw results were analyzed for the extraction of the fluorescence data collected at starting and final temperatures, which were used to calculate a fluorescence difference (ΔF = fluorescence_525nm_ at 14 °C – fluorescence_525nm_ at 25 °C). The analysis was performed by computing ΔF/F0, where F0 is the fluorescence signal at the starting temperature (25 °C). IC_50_ (half maximal concentration) curves were generated by fitting the fluorescence data with a sigmoidal curve equation using GraphPad PRISM® software (version 5, GraphPad Software Inc.). All data point determinations were performed in duplicate before a mean value was calculated, and error bars represent the standard error of the mean.

### Manual Patch Clamp Experiment

Sixteen hours before an experiment, cells were seeded onto poly-D-lysine-coated glass slides (150,000 cells each) and placed in 6-well plates in antibiotic-free medium. Immediately before experiments, the coated glass slides with seeded cells were washed five times with patch-clamp extracellular solution (145 mM NaCl, 10 mM EGTA, 10 mM HEPES, 10 mM Glucose, pH 7.4 with NaOH) and then placed into the recording chamber.

The electrophysiology study involved a whole-cell voltage clamp and intracellular solution consisting of 128 mM CsCl, 10 mM EGTA, 0.7 mM CaCl_2_, 3 mM MgCl_2_, 10 mM HEPES, and 5 mM Na_2_ATP at pH 7.2 with CsOH. For the pulse protocols, cells were held at +40 mV, and outward currents were acquired in uninterrupted recording mode. Data were recorded in standard whole-cell voltage experiments performed at room temperature. For data acquisition and further analysis, an EPC10 digitally controlled amplifier was utilized in combination with PATCHMASTER software (HEKA Electronics, Lambrect, Germany). The EPC10 provides automatic subtraction of capacitive currents by means of the prepulse. The data were filtered at 2.9 KHz (−3 dB, 4-pole Bessel lowpass) and digitized at 100 µs per point. Liquid junction potential showed no correction. The input resistance of the patch pipettes was 2–5 MOhm, and the series resistances were carefully compensated up to 80%. Outward currents elicited by the addition of 50 μM cooling agent 10 or of cooling agent 10 (50 μM) plus compound 1 (300 nM) were recorded.

### *In vivo* experiments in isovolumetric bladder rat model

To assess the activity of compound 1 in preclinical models of painful urologic syndromes as OBS we used an isovolumetric rat bladder animal model. A preliminary PK/ADMET study was conducted to assess the behaviour of compound 1 *in vivo* (Table S3).

Female Wistar rats (190–250 g) obtained from Charles River Laboratories were housed according to animal welfare guidelines. Rats were housed in a specific-pathogen-free environment under strictly controlled light cycle conditions, and food and water were provided ad libitum. All experimental protocols were carried out by Urosphere in accordance with the European Community Council Directive 86/609/EEC. They were performed in accordance with French legislation concerning the protection of laboratory animals and they were approved by French Ministry for Agriculture and Fisheries which provided a currently valid license for experiments on vertebrate animals to Dr. Philippe Lluel.

For intravenous administration, compound 1 was diluted in vehicle (10% of 2:1 solutol:NMP, 16% buffer, and 74% H_2_O) according to body weight to obtain doses of 10 mg/kg. For intravesical administration, compound 1 was diluted at a concentration of 7.56 mg/ml in a solution of 7% 2:1 solutol:NMP, 11.2% buffer, and 81.2% H_2_O. Eight rats were used for the control group and eight were used for the treated group.

Rats were anesthetized with urethane (1 g/kg, i.p.), given as a divided dose of 80% initially and 20% 15 min later. After abdominal incision, the ureters were ligated and sectioned. A catheter was placed into the bladder through the urethra. Then, the urethra was ligated at the level of the urinary meatus. For cystometric experiments with intravenous compound 1 administration a bladder catheter was connected via a T-tube to a strain gauge, which was used to measure the intravesical pressure. Isovolumetric bladder contractions were induced by stepwise injections of physiological saline at room temperature (100 μl every 5 min) until stable RBC occurred. After stabilization and a control period of at least 30 min, characterized by stable and reproducible bladder contractions (basal values), the test substance or vehicle was administered by intravenous infusion (1 mL within 5 min). The effects of compound 1 on micturition frequency (MF) (peaks/30 min), time of inhibition^60^, and threshold volume (ml) (volume inducing RBC) were analyzed. The drug effects were evaluated from the end of the administration up to 1 hour after administration. For intravesical administration, compounds were administered intravesically 3 × 100 μl every 5 min. Then, the bladder was filled with 100 μl of saline every 5 min until the occurrence of rhythmic bladder contraction (RBC) with a maximal volume of 3 mL. When RBC appeared, the intravesical pressure was followed over the next 90 min. Statistical analyses were performed using GraphPadPrism® 4.02. The results are presented as the mean values ± standard error of the mean^58^. For all tests, p < 0.05 was considered statistically significant.

For the intravenous administration results, basal values corresponded to the 30-min period before administration. Basal values of compound 1-treated groups and corresponding vehicle groups were compared using one-way ANOVA followed by Bonferroni test. One-way ANOVA with repeated measures followed by Bonferroni test was used to compare MF basal values with MF values post-administration in each group. Inhibition time and threshold volume in compound 1-treated and corresponding vehicle groups were compared using an unpaired Student’s t-test. For the intravesical administration results, MF values of compound 1-treated and corresponding vehicle groups were compared using two-way ANOVA followed by the Bonferroni test. Threshold volume values obtained in each group were compared using one-way ANOVA followed by the Bonferroni test.

## Results

### SMARTS-based (ligand-based approach) and Docking-based (structure-based approach) virtual screenings

Since both ligand-based and structure-based approaches are limited by their own characteristics we explored the possibility to use a combination of them to obtain a better search for TRPM8 inhibitors in *in silico* screening of the above cited proprietary library.

The SMARTS strings encoding the derived pharmacophore and those designed to filter undesired moieties were used to screen a corporate library of 124,107 compounds^45^ (see Supporting Information for details). As reported in Table 1, this analysis resulted in the selection of 6,400 molecules (20 × 384-well plates, 320 compounds each plate), and the subsequent HTS analysis revealed that this selected subset included 479 confirmed hits (i.e., with a response score > 50).

**Table 1:** Major results for the here performed virtual screening campaigns.

Method	Compounds	Active	Hit rate	EF	Active Scaffolds (AS/Compounds)
Total	11725	1030	8.78	6.27	483 (0.47)
Ligand-based	6400	479	7.48	5.34	238 (0.49)
Structure-based	6400	677	10.57	7.55	282 (0.42)
Consensus	1075	126	11.72	8.37	37 (0.29)

The enrichment factors refer to a random hit rate equal to 1.40% as obtained by previous HTS campaigns on the same target (unpublished data).

Hence, the percentage of confirmed hits was 7.48% with an enrichment factor (EF) of 5.35, which was notable compared with the available random hit rate (1.4%) obtained in previous HTS studies. The ratio of scaffolds *vs*. active compounds of the selected set of compounds was 0.49, which was in line with that obtained by docking simulations (0.42; described further below). This result illustrates that suitably designed SMARTS strings can provide reasonably heterogeneous results that are not heavily constrained by the exploited pharmacophore rules (Table 1). The 10 best-performing SMARTS strings are reported in Table 2; notably, for all the reported strings, the k value was greater than 0, thus confirming that the obtained results were not due to a randomly correct prediction.

**Table 2:** Enrichment Factors (EF) and k values for the 10 SMARTS strings related to the pharmacophoric model (*K values computed as reported by ref.^46^).

SMARTS	Total Ratio	SMARTS Ratio	EF	SMARTS Positive	SMARTS Negative	K Value*
1	0.014	0.4	28.57	2	3	0,391
2	0.014	0.132	9.43	177	1164	0,120
3	0.014	0.127	9.04	10	69	0,114
4	0.014	0.104	7.41	11	95	0,091
5	0.014	0.093	6.66	11	107	0,080
6	0.014	0.088	6.31	25	258	0,075
7	0.014	0.08	5.71	24	276	0,067
8	0.014	0.079	5.67	17	197	0,066
9	0.014	0.063	4.48	14	209	0,049
10	0.014	0.052	3.75	275	4961	0,039

As for docking-based virtual screening, using the protocol described in the methods section, we screened the same corporate library of 124,107 compounds used in SMART-based screening (see Supporting Information for details), and the top-ranked 6,400 compounds were selected (the same number of compounds selected by SMARTS-based filtering). These 6,400 molecules were added to the compounds derived from the ligand based strategy, thus yielding a library of 11,725 diverse compounds that were subsequently analyzed by HTS.

Docking simulations indicated that TRPM8 antagonists simultaneously interact with both Tyr745 and Asp802; hence, the beneficial roles of a suitably spaced hydrogen bond acceptor (HBA), which interacts with Tyr745, and a hydrogen bond donor (HBD) system, which interacts with Asp802 (Fig. 2), were consistent with the conserved pharmacophoric features identified in the proposed ligand-based approach. The first cyclic system should mimic the menthol carbon skeleton, whereas the second aromatic ring should approach Asn799. The interactions of both cyclic systems are strengthened by apolar contacts with the surrounding hydrophobic residues.

As reported in Table 1, there is limited overlap between the subsets selected via ligand-based and structure-based approaches; 1075 compounds are common to both sets (approximately 1/6 of the entire subset). This result suggested that SMARTS strings and docking simulations cover different chemical spaces owing to the different criteria through which the two methods select their molecules. Notably, the docking-based subset included 677 confirmed hits with an enrichment factor of 7.55, which was greater than that achieved by SMARTS strings. Interestingly, and contrary to what was expected, the docking-selected library showed chemical variability, as represented by a ratio of scaffolds per active compound that was comparable to that obtained using SMARTS strings (0.42 vs. 0.49). These results demonstrate that the extension of the pharmacophore overcome the limits of the atom mapping of the SMARTS encoding.

### High-Throughput Screening

To confirm the biological activity of the compounds selected by above described *in silico* screening approaches, we performed a wet-lab HTS analysis of a focused subset of 11,725 compounds. In the primary screen step, all 11,725 compounds were tested once at 10 μM. The screening quality parameter Z′ was evaluated for each plate both in the compound addition phase (CA; when the compound was applied to the cells) and in the subsequent target activation phase (TA; when the reference agonist was added at EC_80_ concentration to open the channel). An assay plate was considered valid in CA if Z′ was greater than 0.4, whereas for TA, Z′ had to be greater than 0.5 (the HTS evaluation strategy is reported in the Supporting Information). Overall, the mean Z′ for TA was 0.77 with a standard deviation of 0.068, whereas for CA, the mean Z′ was 0.88 with a standard deviation of 0.06. Z′ prime calculation is based on the signal distribution of the positive and negative controls (MIN and MAX signals) allocated on fixed well positions on each plate.

Compounds active upon CA (potential agonists) and compounds that exhibited an unusual and rapid increase in the fluorescent signal upon CA (indicating potential auto-fluorescence) were flagged and excluded from further characterization. The threshold for the primary screening hit selection was set at 50% inhibition. The primary screen identified 1727 compounds, corresponding to an impressive hit rate of 14.7%. The selected compounds were next subjected to the hit confirmation phase in which they were tested at three concentrations (1.0, 3, and 10 μM) with triplicate data points. Next, on the basis of performance, a response score was computed for each compound (i.e., a weighted score averaging the inhibition percentage values at the three tested concentrations) and was used to rank the hit confirmation data.

In this way, 1,030 compounds were selected for activity determination (IC_50_ calculations) because they showed response scores >50. During the activity determination assay, full dose-response curves were obtained for eight different concentrations in triplicate. Remarkably, 21 compounds were identified as strong blockers with submicromolar IC_50_ values and entered a lead optimization process. As summarized in Table 1, the hit confirmation assay yielded an overall hit rate of 8.78, corresponding to an enrichment factor of 6.27. This EF was calculated using a random hit rate of 1.40%, a value that was obtained from previous HTS studies of TRPM8.

### Naphthyl derivatives identified as a new class of TRPM8 inhibitors

**Figure 4:**
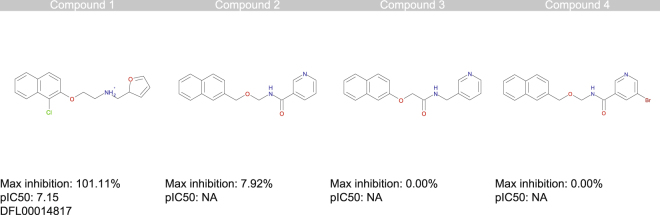
Naphthyl derivatives. Structure and TRPM8 inhibition values for the naphthyl derivatives identified by the HTS campaign (the %inhibition values are obtained at 10 mM).

Among the diverse chemical classes selected by SMARTS-based and docking-based strategies, naphthyl derivatives, resulted in the 21 more active compounds identified from HTS screening We identified four naphthyl derivatives: one naphthyl derivative with an inhibition percentage greater than 50% (compound 1) and three inactive compounds (compounds 2–4) (Fig. 4). Naphthyl derivatives are a previously undescribed chemical class of TRPM8 inhibitors and are interesting because of their drug-likeness, chemical tractability and potency.

Structure-activity relationships can be inferred through ligand-based speculation; however, the high degree of diversity induced by the screening selection criteria prevented the development of a congeneric series (different scaffolds) of the naphthyl compounds, and the multiple points of modification in the structure do not allow clear structure-activity relationships to be derived. Nevertheless, docking simulations using a TRPM8 homology model were performed for the naphthyl compounds, which enabled some characteristics to be elucidated regarding the role of the different functional groups on the activity of the compounds (Figure S2).

Figure 2 shows in detail the putative complex for compound 1 within the TRPM8 binding site, and provides evidence for the key role played by the ionic interaction between the ligand’s ammonium head and Asp802. In detail, the naphthyl moiety engages the hydrophobic sub-pocket normally occupied by menthol, a region framed by Tyr745, Ile746, Leu806 and Phe809, and elicits π-π stacking and hydrophobic interactions with these residues. The positively charged amino group strongly interacts with Asp802 via a salt bridge, and the furan ring is located between Leu750 and Phe794 near the amidic group of Asn799. Interestingly, the inactive naphthyl compounds also locate in the orthosteric binding site similarly to compound 1, substantially contacting the same key residues with a common pattern of interactions. However, the major differences common to all inactive compounds include the substitution of the amine group with amide. These differences led to *i)* the consequent loss of flexibility of the central region of the molecule (bringing the polar groups) and, above all, *ii)* positive charge loss, thus weakening the H-bond with Asp802, which, as demonstrated by single point mutagenesis experiments (see below), is the key residue for the interaction of this class of molecules.

### Pharmacological characterization of compound 1

#### Analysis of binding mode

**Figure 5:**
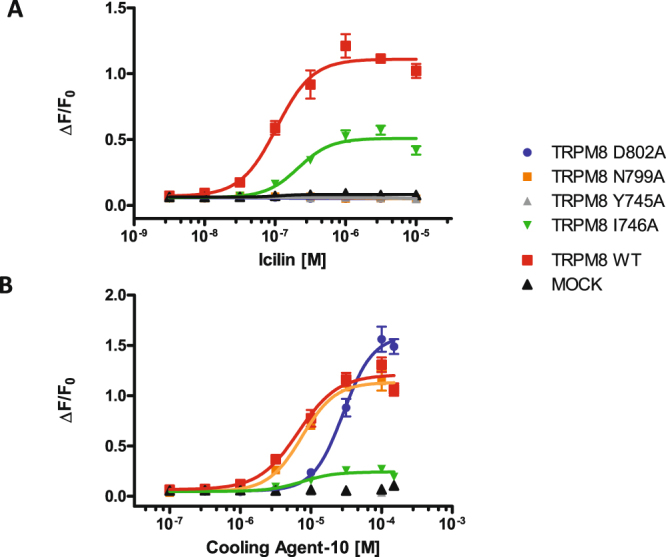
Effects of individual TRPM8 point mutations on calcium responses induced by cooling agent 10 and icilin. Shown data represent mean ± standard error of the mean (SEM) of quadruplicate determinations of a representative experiment (n = 4). Statistical analysis was performed using unpaired Student’s t test with GraphPad Prism (95% confidence interval). (**A**) Dose response curves of icilin on mutant TRPM8 receptors. The substitutions Y745A, D802A and N799A abrogated agonist activity. The I746A substitution significantly (p < 0.05) shifted pIC_50_ of Icilin from 6.6 to 6.9. (**B**) Dose response curves of Cooling Agent 10 on mutant TRPM8 receptors. The substitution I746A strongly reduced agonist activity in term of efficacy while D802A significantly (p < 0.0001) shifted the pIC_50_ of the agonist from 5.1 to 4.5. Other mutations did not have effects on Cooling agent 10 activity.

To investigate the binding mode of our naphthyl TRPM8 antagonist, we generated four different TRPM8 single point mutants. The following amino acid positions were selected to be mutated in alanine in separate constructs: Tyr745, Ile746, Asn799, and Asp802. Asn799 and Asp802 are required for icilin sensitivity in mammalian TRPM8^61^, whereas Tyr745 and Ile746 are the residues involved in activation by menthol and its related molecules, such as Cooling agent 10^49,62,63^. Activities of icilin and menthol-related molecule Cooling agent 10 were evaluated on appropriate mutant set (Fig. 5A and B). Data obtained from at least two separate experiments were used to calculate the icilin and Cooling agent 10 EC_80_ values for each TRPM8 mutant to properly activate them in the subsequent antagonist-challenge experiments.

As expected, for the Tyr745 and Ile746 mutants, it was not possible to obtain good activation nor by Cooling agent 10 neither by icilin^62,63^ (Fig. 5A and B); the Tyr745 mutation completely abolished the activation, whereas with Ile746, minimal residual activation was still detected. Moreover, mutations of Asn799 and Asp802 in Ala completely abolished TRPM8 activation by icilin, thus indicating that these amino acids are necessary for icilin binding^61^ (Fig. 5A); however, these mutations did not affect either maximal TRPM8 activation or EC_50_ in TRPM8 activation by Cooling agent 10 (Fig. 5B). On the basis of these results, the activity of compound 1 was evaluated for TRPM8 with mutations of the following residues: Ile746 (to assess its ability to inhibit icilin-mediated activation), and Asn799, Asp802, and Ile746 (to test its ability to inhibit Cooling agent 10 activation).

**Figure 6:**
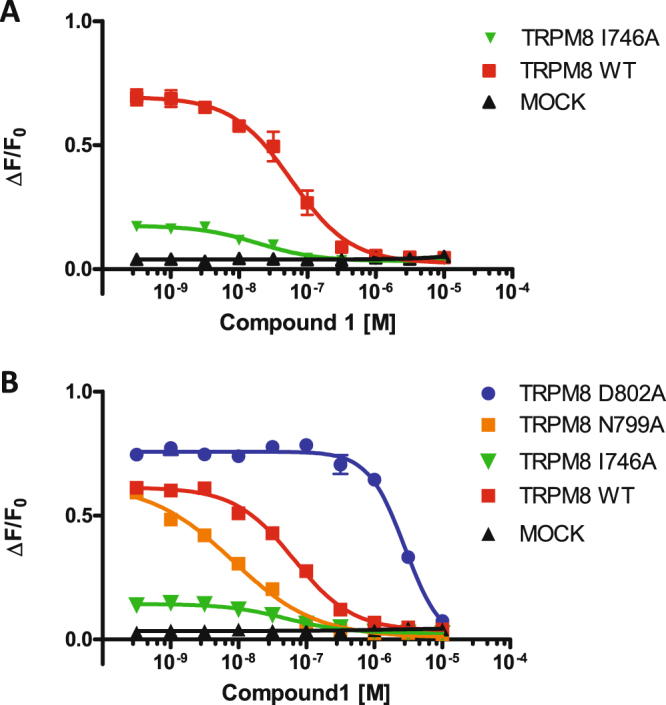
Effects of individual TRPM8 point mutations on Compound 1-mediated inhibition of calcium responses. Mutant and wt TRPM8-transfected cells were exposed to increasing concentrations of Compound 1 and then stimulated with icilin (**A**) or Cooling agent 10 (**B**) at the specific EC80 concentration. Shown data represent mean ± SEM of quadruplicate determinations of a representative experiment (n = 2). Statistical analysis was performed using unpaired Student’s t test with GraphPad Prism (95% confidence interval). (**A**) Dose response curves of Compound 1 activity on Icilin activation. The substitution I746A significantly (p = 0.003) reduced compound 1 inhibition shifting pIC50 from 7.2 to 7.7. (**B**) Dose response curves of Compound 1 inhibition of Cooling Agent 10 activation of TRPM8 receptor. The substitution D802A shifted its pIC50 from 7.2 to 5.5 significantly decreasing (p < 0.0001) Compound 1 potency, while N799A significantly increase Compound 1 potency (p < 0.0001) shifting pIC50 from 7.2 to 8.0.

For the icilin-mediated TRPM8 activation inhibition, the Ile746 substitution significantly (p-value < 0.05) shifted the pIC_50_ of compound 1 from 7.2 to 7.7 (Fig. 6A). By contrast, two different phenomena were detected in inhibiting the activation of cooling agent 10 (Fig. 6B). First, Asp802 substitution markedly affected the inhibition efficacy of compound 1, shifting its pIC_50_ from 7.1 to 5.5, almost a 2-fold decrease; second, Asn799 mutation enhanced the potency of compound 1 from a pIC_50_ of 7.1 to 8. If the former effect was expected, the latter effect was surprising, because only the K856A mutation has been reported result in a gain of function^64^. In this case, the mutation appears to affect the TRPM8 channel sensitivity in chemical and physically related activation.

A possible explanation for the effect of Asn799 was found by examining the results from the docking experiments and homology modeling. In contrast to Asp802, whose carboxylic function clearly interacts with the amine of the ligand, the Asn799 side chain does not bind directly to compound 1. Additionally, docking studies suggested that, in the resting state, Asn799 engages the nearby Asp802 side chain with an intramolecular hydrogen bond; thus, the alanine mutation, by not allowing this interaction, makes Asp802 more available for the interaction with compound 1.

Compound 1 was also tested in schild regression experiments^65^ and compared with icilin and cooling agent (data not shown) to better characterize its mechanism of action. In these experiments, seven points for dose-response curves with icilin at seven different concentrations were analyzed. Schild regression analysis of compound 1 versus icilin suggested an orthosteric binding mechanism, causing a right shift of the icilin dose-response curves at increasing concentrations of antagonist (data not shown).

These experiments demonstrated that compound 1 binds in the ligand binding pocket of TRPM8 contacting Ile746, Asp802 and Asn799. Since we mutated the pocket in which physiological agonists of TRPM8 are described to bind and shild regression experiments revealed a right shift of the icilin dose-response curves, we supposed that compound 1 act as an orthosteric inhibitor.

### Inhibitory activity of compound 1

In HTS compound 1 resulted a potent inhibitor of wild type TRPM8 so we analyzed its inhibitory behavior in deeper details performing calcium mobilization assays in dose-response with different TRPM8 agonists as icilin, cooling agent 10 or cold. Moreover, we also analyzed the inhibitory activity of compound 1 in experiments with different read out as patch clamp assay.

**Figure 7:**
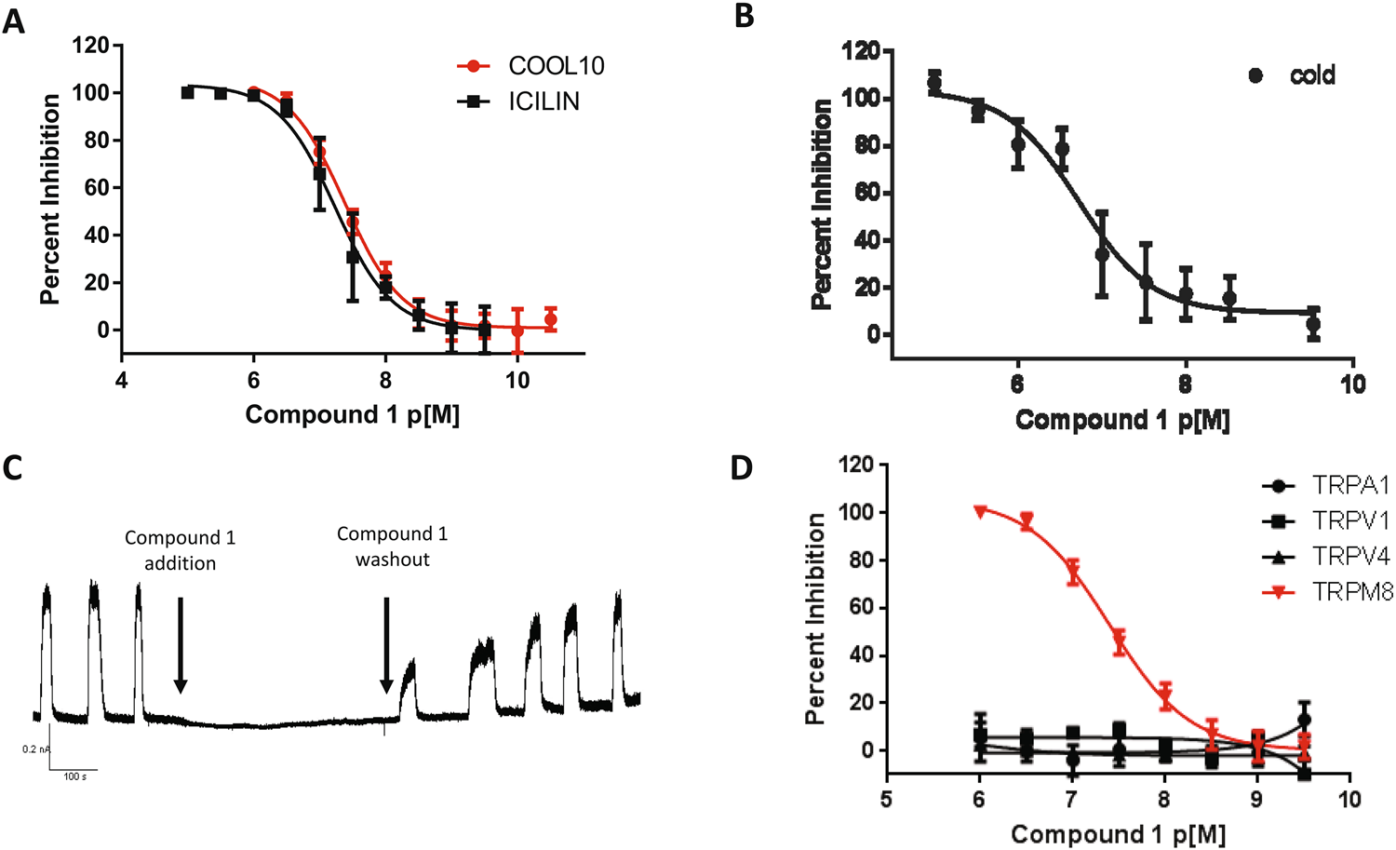
Validation of TRPM8 antagonist compound 1 by orthogonal assays and compound selectivity. (**A**) Seven points dose-response curves of compound 1 inhibition of cooling Agent 10 and Icilin-induced TRPM8 activation using an intracellular calcium mobilization assay. Compound 1 pIC50s against cooling agent 10 and icilin are similar (pIC50 7.38 and 7.23 respectively). Shown data represent the mean ± SEM (error bars) of triplicate determinations of a representative experiment (n = 2). (**B**) Dose-response curve of compound 1 tested by using cold temperatures as stimulus. Compound 1 pIC50 against cold is similar to that calculated for chemical agonists (pIC50 6.73). Shown data represent the mean ± SEM (error bars) of triplicate determinations of a representative experiment (n = 2). (**C**) Test of compound 1 activity at manual patch clamp. Outward currents were elicited upon addition of cooling agent 10 at + 40 mV, in the presence and in the absence of compound 1. Compound 1 addition completely abrogated electrical impulse transmission. (**D**) Compound 1 selectivity towards TRPM8 related receptors TRPA1, TRPV1, TRPV4. Compound 1 is completely inactive against all TRPM8 related receptors tested (p < 0.001). Shown data represent the mean ± SEM (error bars) of triplicate determinations of a representative experiment (n = 2).

Compound 1 was found to be a potent inhibitor of TRPM8, showing activity in the nanomolar range in the calcium mobilization assay regardless of the agonist used (pIC_50_ value of 7.38 with Cooling agent 10 and 7.23 with icilin) (Fig. 7A).

To further validate the TRPM8 blocking behavior of compound 1 we analyzed its behavior towards the physiological stimulus of TRPM8: cold. Increasing concentrations of compound 1 were incubated with HEK-293/hTRPM8 cells exposed to a temperature decrease from 25 to 14 °C, and Ca^2+^ mobilization was recorded with a Ca^2+^-sensitive fluorescent dye. In this physiological stimulus assay, compound 1 inhibited TRPM8 activation (Fig. 7B) with a pIC_50_ of 6.76.

In a second orthogonal assay, TRPM8 inhibition by compound 1 was evaluated by manual patch-clamping. Outward currents were elicited through the addition of Cooling agent 10 at +40 mV in the presence and absence of compound 1. When compound 1 was perfused, the outward current was almost completely blocked, thus suggesting a decrease in TRPM8 activity (Fig. 7C). As expected, after compound 1 was washed out, TRPM8 activity was restored.

Remarkably, compound 1 also showed an excellent selectivity profile toward other members of the TRP channels family, because it was completely inactive on TRPA1, TRPV1 and TRPV4 (Fig. 7D). The three channels selected, TRPV1, TRPA1 and TRPV3, have an integrated functional role in common with TRPM8. Moreover, TRPV1 and TRPA1 have been associated with side effects including burning and hot flushing.

In conclusion, we demonstrated the inhibitory activity of compound 1 with three different stimuli (icilin, cooling agent 10 and cold) and two orthogonal assays (calcium mobilization and manual patch clamp). With all stimuli, the IC_50_ of compound 1 were similar. Additionally, we demonstrated the selectivity of compound one on clinically relevant related receptors TRPA1, TRPV1 and TRPV4.

### *In vivo* efficacy of compound 1 in an overactive bladder rat model

To evaluate the potential of compound 1 in the treatment of urologic painful syndromes TRPM8-related we tested its activity in an isovolumetric rat bladder model analyzing its effect on MF, inhibition time and threshold volume with or without intravenous or intravesical administration of compound 1.

**Figure 8:**
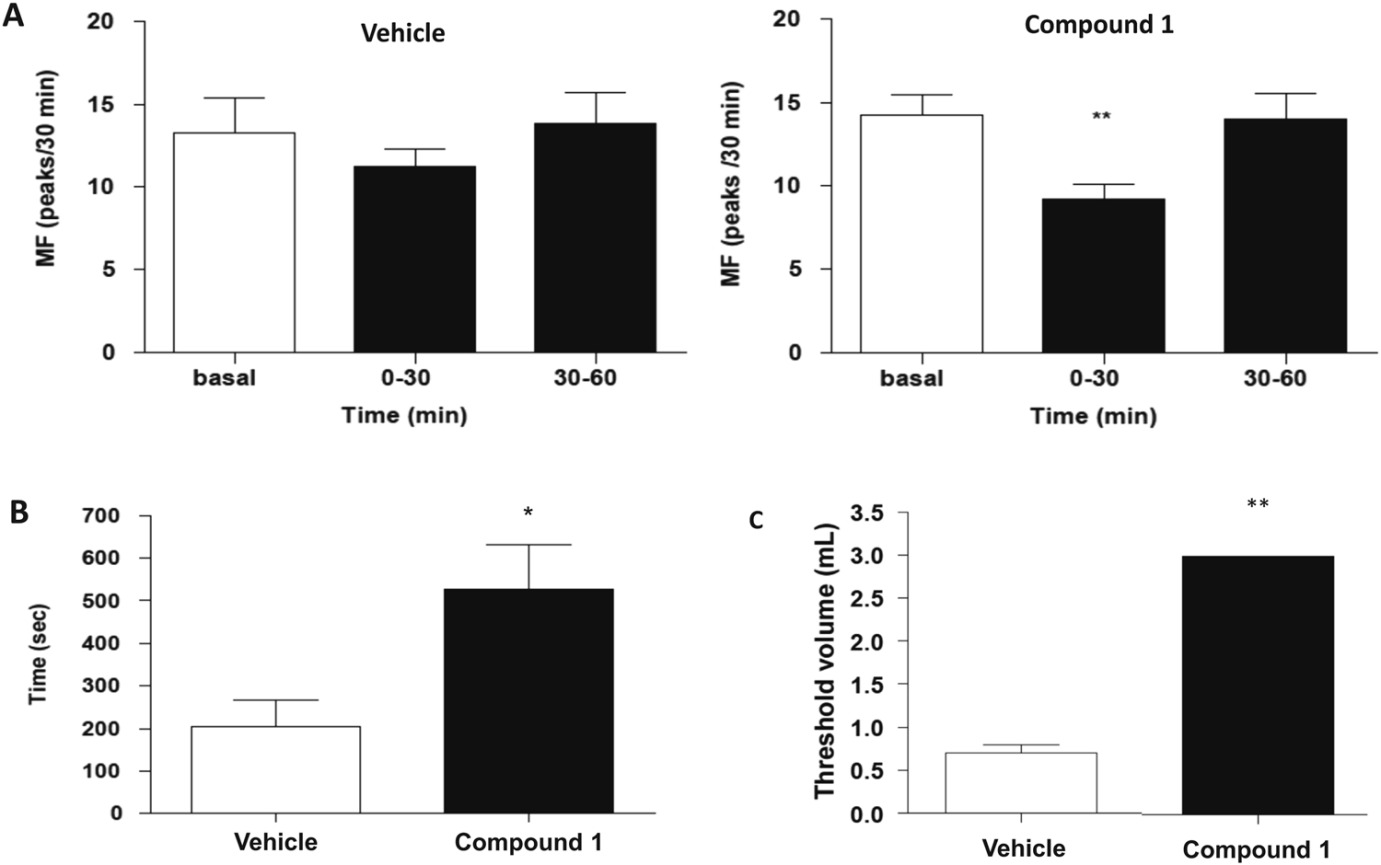
*In vivo* effects of compound 1 in isovolumetric bladder rat model. The results were given as mean values ± standard error of the mean (sem) of the measurement in 8 different rats. (**A**) Effects of intravenous administration of vehicle (on the left) and compound 1 at 10 mg/kg (on the right) on micturition frequency (MF). No significant changes were observed upon vehicle administration (on the left), while compound 1 (on the right) significantly reduced MF at 30 min (p < 0.01). (**B**) Effects of intravenous administration of vehicle and compound 1 at 10 mg/kg on inhibition time. Compound 1 significantly enhanced inhibition time (p < 0.05). (**C**) Effects of intravescical administration of vehicle and compound 1 at 2.268 mg. Compound 1 significantly increased (p < 0.01) the threshold volume inducing RBC.

When MF basal values of vehicle-treated and compound 1-treated groups were compared, no significant difference between the values was observed (Fig. 8A). Compound 1 significantly decreased MF 0–30 min post-administration (p < 0.01; one-way ANOVA with repeated measures followed by Newman-Keul test) (Fig. 8A). However, no effect was observed at 30–60 min post-administration. Intravenous administration of vehicle did not modify MF. Additionally, after compound 1 administration, the inhibition time of RBC was significantly higher than the inhibition time observed after vehicle administration (526 ± 106 versus 204 ± 63 sec; p < 0.05; unpaired Student’s t-test) (Fig. 8B). No significant difference between threshold volumes in these two groups was observed.

Intravesical administration of compound 1 (2.268 mg) abolished the occurrence of RBC. Indeed, the maximal volume of filling (3 mL) was reached without the occurrence of RBC. Compared with vehicle, the threshold volume after compound 1 administration was significantly higher (3 mL versus 0.70 ± 0.09 mL) (Fig. 8C).

In conclusion, compound 1 slightly reduced RBC frequency when it was administered by the intravenous route but abolished the occurrence of RBC when it was administered intravesically. These results suggest that this TRPM8 receptor antagonist inhibits the afferent pathway of the bladder.

## Discussion

Although more than 100 papers on the TRPM8 ion channel have been indexed in PubMed in the last year, thus revealing the strong interest of the scientific community regarding the role of TRPM8 in many pathological conditions, there is a lack of candidate drugs for further investigation of the potential use of TRPM8 modulators in the clinic.

Notably, most of the available TRPM8 antagonists exhibit an unsuitable selectivity profile, which hampers their clinical applications; consequently, very few compounds have advanced to clinical trials^66,67^. Only three compounds, PF-05105679^66^ (ClinicalTrials.gov Identifier: NCT01393652) from Pfizer, AMG-333 (ClinicalTrials.gov Identifier: NCT01953341) from Amgen, and the cannabinoid Cannabidivarin (GWP-42006) (ClinicalTrials.gov Identifier: NCT02365610) from GW Pharmaceuticals, have entered clinical trials. However, PF-05105679 was discontinued because of side effects, and results of the AMG-33 trial have not yet been disclosed even though the study ended in September of 2014. Given this landscape, the naphthyl analogs reported here may provide a good balance of potency and selectivity, thus decreasing unfavorable off-target effects and representing valuable candidates for clinical studies.

Here we used two virtual screening approaches in combination: one ligand-based approach using SMARTS strings as a pharmacophoric filter and one structure-based approach using molecular docking techniques on a validated homology model to identify a novel class of TRPM8 inhibitors with clinics suitable characteristics. Analysis of the screening results highlighted the beneficial effects of combining ligand- and structure-based methods. As detailed in Table 2, the compounds identified by both methods included 126 confirmed hits, thus yielding an enhanced enrichment factor of 8.4. Hence, the two methods, while covering a diverse chemical space, can be successfully combined in a parallel approach, thereby allowing the selection of only compounds able to form the most stable complexes from among the compounds fulfilling the pharmacophore filters. Ligand-based approach are limited by the selection of compounds used to derive the pharmacophore, so is likely that with this approach it would be found molecules similar to those selected at the beginning, on the other hand structure-based approach is limited by a rigid conformation of the receptor which selected only molecules which would fit in such rigid structure. A combined approach covers a wide chemical space and can identify different chemical classes of TRPM8 inhibitors.

With the described combined *in silico* approach, we identified naphthyl derivatives as a promising novel chemical class of TRPM8 inhibitors. To date, approximately 120 different chemotypes can be enumerated among all known TRPM8 antagonists^47^. These chemotypes include the following examples: benzyloxybenzene derivatives, such as AMTB (Bayer)^68^, Cpd 87 (Glenmark, WO2010010435) and Cpd 14 (Pfizer)^69^; arylamides, arylsulfonamides and close analogs, such as RQ-00203078^705047^ (RaQualia)^51^ and Cpd 7 s (Janssen)^70^; AMGEN tetrahydroisoquinolines derivatives^71^; chromanes and chromenes, such as Cpd 8f (Glenmark)^24^ and Cannabichromene (GW Pharmaceuticals)^72^, respectively; azetidin-2-ones^23^; and diphenylpyrazoles (Kissei, WO2016208602) Napththyl derivatives have the proper chemical characteristics and tractability to overcome the lead optimization process. The compound 1 lead presented in this paper demonstrated to be potent and selective before any chemical optimization. Moreover, in *in vivo* proof of concept compound 1 had adequate PK/ADME properties and presented a positive outcome in approaching the treatment of painful syndromes in urologic field, thus supporting the further optimization of this compound.

It is noteworthy that compound 1 didn’t have any effect on inhibition of three selected TRP channels, namely TRPV1, TRPA1 and TRPV3. The three channels selected for the selectivity study share with TRPM8 an integrated role in pain, inflammation, and cancer^14^, and they can be co-regulated by prolactin^4,10,73^. TRPV1, TRPA1, TRPV3 and TRPM8 are expressed in distinct populations of primary afferent neurons and have been demonstrated to mediate hypersensitivity to thermal, mechanical and chemical stimuli after tissue and nerve injury and inflammation^11,74,75^. Most TRPV1 neurons also express TRPA1, whereas TRPM8 neurons are different from TRPV1/TRPA1 fibers^76^. The selected receptors have been demonstrated to play a pivotal role in nociception and hence are potential targets of new analgesic therapeutics; however, ligands are often poorly selective toward these TRP receptors. Additionally, because TRPV1 and TRPA1 have been associated with side effects such as burning sensations, the reported side effects because of which PF-05105679 has been retired from clinical testing^77–80^, enhanced selectivity toward these receptors may provide a good starting point for avoiding these adverse effects.

In conclusion, we present a case study of the benefits of a combined *in silico* approach to increase the enrichment factor of screening campaigns for new lead compounds. Our approach identified a new class of potent and selective TRPM8 inhibitors belonging to the class of naphthyl derivatives that may be developed as clinical candidates because of their favorable pharmacological profiles (potency, efficacy, selectivity and chemical tractability).

## Electronic supplementary material

Supporting information
