# Publisher Correction: Novel selective, potent naphthyl TRPM8 antagonists identified through a combined ligand- and structure-based virtual screening approach

**DOI:** 10.1038/s41598-018-21902-z

**Published:** 2018-03-06

**Authors:** Andrea R. Beccari, Marica Gemei, Matteo Lo Monte, Nazareno Menegatti, Marco Fanton, Alessandro Pedretti, Silvia Bovolenta, Cinzia Nucci, Angela Molteni, Andrea Rossignoli, Laura Brandolini, Alessandro Taddei, Lorena Za, Chiara Liberati, Giulio Vistoli

**Affiliations:** 1 Dompé Farmaceutici SpA, Via Campo di Pile, L’Aquila, L’Aquila Italy; 2 Axxam S.p.A, Via Meucci, 3, I-20091 Bresso, Italy; 3 Dipartimento di Scienze Farmaceutiche, Università degli Studi di Milano, Via Mangiagalli, 25, I-20133 Milano, Italy; 4 Joint Bioinformatics Group, Institute of Protein Biochemistry, National Research Council, Via P. Castellino 111, Napoli, Italy

Correction to: *Scientific Reports* 10.1038/s41598-017-11194-0, published online 08 September 2017

In the original version of this Article, the author Matteo Lo Monte was incorrectly indexed. This error has now been corrected.

