# Supplementary material for: Novel selective, potent naphthyl TRPM8 antagonists identified through a combined ligand- and structure-based virtual screening approach
Source: Sci Rep. 2017 Sep 8;7:10999. doi: 10.1038/s41598-017-11194-0 (PMC5591244; doi:10.1038/s41598-017-11194-0)
Supplement: Supplementary file 1 — Supporting information [file 41598_2017_11194_MOESM1_ESM.doc]

**Novel selective, potent naphthyl TRPM8 antagonists identified through a combined ligand- and structure-based virtual screening approach**

Andrea R. Beccari^ad^*, Marica Gemei^a^*, Matteo Lo Monte^d^, Nazareno Menegatti^b^, Marco Fanton^a^, Alessandro Pedretti^c^, Silvia Bovolenta^b^, Cinzia Nucci^b^, Angela Molteni^b^, Andrea Rossignoli^b^, Laura Brandolini^a^, Alessandro Taddei^b^, Lorena Za^b^, Chiara Liberati^b^, and Giulio Vistoli^c^

a) Dompé farmaceutici S.p.A. Research Centre, Dompé farmaceutici S.p.A, Via P. castellino 111, Napoli, Italy

b) Axxam S.p.A, Via Meucci, 3, I-20091, Bresso, Italy

c) Dipartimento di Scienze Farmaceutiche, Università degli Studi di Milano, Via Mangiagalli, 25, I-20133 Milano, Italy

d) Joint Bioinformatics Group, Institute of Protein Biochemistry; National Research Council, Via P. Castellino 111, Napoli, Italy

Supplementary information:

Library generation.

HTS Evaluation

Figure S1

Table S1

Table S2

Additional references

**Library generation.**

The compound collection used for the in silico screening is composed by 124,107 synthetic small molecules and is a joined Dompé and Axxam corporate collections. For the assembly of the screening collection several computational tools were used: millions of commercially available compounds were screened in silico, applying a number of scientifically recognized filters to select a set of compounds that adhere to strict drug-like criteria: almost 90% of the library has zero violations of Lipinski’s Rule of Five (1) for oral drug-likeness and more than 85% has zero violations of Oprea’s parameters for lead-likeness (2). Then, several other filters were applied to minimize the number of known aggregators (3-4), false positives (5), toxicophores (6), and compounds containing reactive functional groups (7). Particular attention was given to the choice of compounds with calculated favorable physicochemical and pharmacokinetic (ADMET) parameters, resulting in almost 80% of the library having predicted oral drug-like properties and no major liabilities (8-11). The selection of compounds with a relatively high degree of novelty was performed by application of a modified version of the framework analysis proposed by Murcko (12). Following a hierarchical fragmentation schema, progressive levels of structure abstractions were used to classify compounds according to their scaffolds or frameworks. Compounds were then clustered by framework or scaffold and compared to similarly clustered compounds currently at various stages of development, including launched drugs. The library was assembled in such a way that frameworks and scaffolds not represented in development compounds make up 63-71% of the library, respectively. Cluster analysis of these structural descriptors was also applied to ensure high chemical diversity of the screening collection.

**HTS Evaluation**

In the implemented workflow, the steps of data analysis can be schematized as follows: (i) import of FLIPR TETRA files (Seq1) without corrections (the data from the original kinetic file is divided into two distinct phases, named respectively as Compound Addition (CA) and Target Activation (TA)); (ii) calculate Normalization Factor (NF) as Baseline Mean / Baseline; (iii) calculate the Kinetic Response Value (KRV) of the profile for TA phase as follows:

TA_KRV = NF*(Max(TP_113..TP_171) – Average (TP_110..TP_112))

(iv) calculate the Kinetic Response Value (KRV) of the profile for CA phase as follows:

CA_KRV = NF*(Max(TP_011..TP_103) – Average (TP_005..TP_007))

Compute Response Value for Target Activation (CA) Phase:


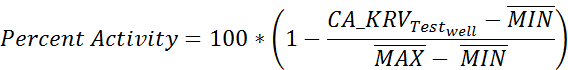


Compute Response Value for Compound Addition (TA) Phase:


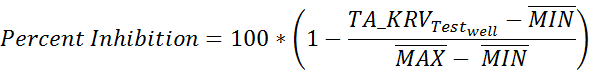


Compute Z’:


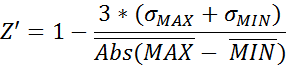


Figure S1: the 12 structurally diverse TRPM8 antagonists selected for pharmacophore development,

**Table S1:** agonists and antagonists concentration scheme for intracellular calcium mobilization-based assays

| **Experiment** | **Agonist [C]** | **Antagonist [C]** |
| --- | --- | --- |
| HTS primary screening | Cool 10: 30μM | 10μM |
| HTS hit confirmation | Cool 10: 30μM | 1, 3, 10 μM |
| IC50 determination Dose-response | Cool 10: 30μM  Icilin: 650nM | Compound 1 Vs COO10 (10 concentrations half log increment): From 3.16*10-5 μM to 1 μM  Compound 1 Vs ICILIN (10 concentrations half log increment): From 3.16*10-4 μM to 10 μM |
| Mutants activity  H720A, Y745A, I746A, D802A, WT, MOCK | Cool 10: 30, 30, 480, 120, 30, 30μM  Icilin: 350, na, 650, na, 650, 650nM | (10 concentrations half log increment): From 3.16*10-4 μM to 10 μM |
| Selectivity assays | For TRPA1: AITC 10 microM  For TRPV1: Capsaicin 100 nM  For TRPV4: GSK1016790A 20 nM  For TRPM8: Cool10 30μM | (8 concentrations half log increment): From 9.5pM to 6pM |

Table S2 Collection of published compounds (MDDR Database) was analyzed in order to identify moieties directly connected to mechanisms of action. With the same rational used for the definition of pharmacophore strings, a selection of 7 SMARTS strings were presented. Through a sub-structural search, they were used to eliminate cross-selective compounds from the library.

| SMARTS Strings | Mechanism Of Action |
| --- | --- |
| [#7,#8](-[#6](-[#6]-[#6](-[#7,#8]-[*R,#6!R])=[#8])=[#8])-[*R,#6!R] | Antineoplastic |
| [c,n]1[c,n][c,n]([c,n][c,n]1)-[#6]-[#6](=[#8])-[#7]-[*R,#6!R] | Antineoplastic |
| [#16](=[#8])(=[#8])(-[#7]-[#6](-[#6]-[#7]-[*R,#6!R])=[#8])-[*R,#6!R] | Anticoagulant |
| [#7&R0](-[#6&R0](-[#6&R0](-[#7&R0]-[*R,#6!R])-[*R])=[#8])-[*R,#6!R] | Anticoagulant |
| [#6](-[#6](-[#7]-[*R,#6!R])=[#8])(-[#7]-[*R,#6!R])=[#8] | Antineoplastic |
| [#6](-[*R])(=[#8])-[#7]-[#6](-[#6](-[#6]-[#7]-[*R,#6!R])-[#8])-[#6]-[*R] | Nootropic Agent |
| [*R,#6!R]-[#7!R]-[#6!R]-[#6!R](-[#7!R]-[*R,#6!R])=[#8] | Anticoagulant |

The Enrichment Factor (E.F.) obtained using the strings were calculated by the following equation:


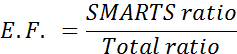


where:


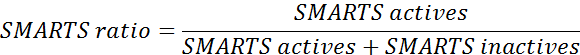


and:


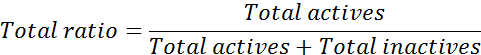


**Figure S2: TRPM8-Compound 3 complex, detail of the binding site**

A) Top view and B) side view of the binding site of TRPM8 in complex with inactive Compound 3. Compound 3 well fits into the “menthol-hosting” sub-pocket, where it elicits π-π stacking and hydrophobic interactions with Tyr745, Ile746 and Leu806.  An H-bond between the amidic hydrogen and Asp802 is preserved, though the loss of a positive charge (present in active compounds) reduces its strength. Locating few Armstrong more towards the extracellular edge of the membrane, Compound 3 loses the π-π interaction with Phe794, approaching only Leu750 with the 3-pyridyl group. This is in line with the pharmacophore, according to which both aromatic tails are important.

ADDITIONAL REFERENCES

Lipinski CA, Lombardo F., Dominy BW, Feeney PJ, Experimental and computational approaches to estimate solubility and permeability in drug discovery and development settings, Adv. Drug Del. Rev., 1997, 23, 3-25

Oprea TI, Property distribution of drug-related chemical databases, J. Comp. Aid. Mol. Des. 2000, 14, 251

Feng BY, Shelat A, Doman TN, Guy RK, Shoichet BK, High-throughput assays for promiscuous inhibitors, Nat Chem Biol 2005, 1, 146-8.

Feng BY, Simeonov A, Jadhav A, Babaoglu K, Inglese J, Shoichet BK, Austin CP, A high-throughput screen for aggregation-based inhibition in a large compound library, J Med Chem 2007, 50, 2385-90

Pearce BC, Sofia MJ, Good AC, Drexler DM, Stock DA, An empirical process for the design of high-throughput screening deck filters, J Chem Inf Model 2006, 46, 1060-8

Kazius J, McGuire R, Bursi R, Derivation and validation of toxicophores for mutagenicity prediction, J. Med. Chem, 2005, 48, 312-320

Metz JT, Huth JR, Hajduk PJ, Enhancement of chemical rules for predicting compound reactivity towards protein thiol groups, J Comput Aided Mol Des 2007, 21, 139-44

Muegge I, Selection criteria for drug-like compounds, Med Res Rev 2003, 23, 302-21

Olah M, Bologa CG, Oprea TI, Strategies for compound selection, Curr Drug Discov Technol 2004, 1, 211-20

Walters WP, Murcko MA, Prediction of 'drug-likeness', Adv Drug Deliv Rev 2002, 54, 255-71

Bemis GW, Murcko MA, The properties of known drugs. 1. Molecular frameworks. J Med Chem 1996, 39, 2887-93

SMARTS Teory Manual, Daylight Inc. <http://www.daylight.com/dayhtml/doc/theory/theory.smarts.html>
